# The use of rumen-protected amino acids and fibrous by-products can increase the sustainability of milk production

**DOI:** 10.3389/fvets.2025.1588425

**Published:** 2025-09-08

**Authors:** Damiano Cavallini, Martina Lamanna, Riccardo Colleluori, Simone Silvestrelli, Francesca Ghiaccio, Giovanni Buonaiuto, Andrea Formigoni

**Affiliations:** Department of Veterinary Medical Sciences, University of Bologna, Ozzano dell’Emilia (BO), Bologna, Italy

**Keywords:** dairy nutrition, fiber-rich co-products, rumen-bypass amino acids, sustainable farming, feed efficiency

## Abstract

Optimizing the balance between dietary proteins and energy in dairy cow feeding is key to improving milk production efficiency and sustainability, with current strategies focusing on the inclusion of fibrous by-products as alternatives to cereals and the reduction of protein content through the use of rumen-protected amino acids (AA), thereby reducing competition with human food resources. This study involved 8 multiparous Holstein cows assigned to one of four isoenergetic diets: High Protein and High Cereals (HP-HC, 15% CP; 27% starch), High Protein and High Fibrous By-products (HP-HF, 15% CP; 20% starch), Rumen-Protected Amino Acids and High Cereals (AA-HC, 12% CP; 26% starch), and Rumen-Protected Amino Acids and High Fibrous By-products (AA-HF, 12% CP; 20% starch). The trial used a Latin square design, with data collected on intakes, milk production, rumen fermentation, nitrogen (N) utilization, diet digestibility and feaces residual nutrients analysis. The results showed that the ration based on fibrous by-products did not affect DMI and rumination time. Cows fed with lower protein sources and AA had significant lower ruminal ammonia levels (−1.61 mg/dL), improved N utilization efficiency (+5.61%) and reduced water intake (−21 L/day). These findings suggest that formulating rations substituting cereals with fibrous by products and reducing the N intake using rumen protected AA improve N efficiency and reduce the water consumption enhancing the environmental sustainability of milk production. Milk production and fiber digestibility were greatest in HP-HC diet indicating that some refinements of this ration strategy are needed to maintain animal performances.

## Graphical abstract

**Figure:**
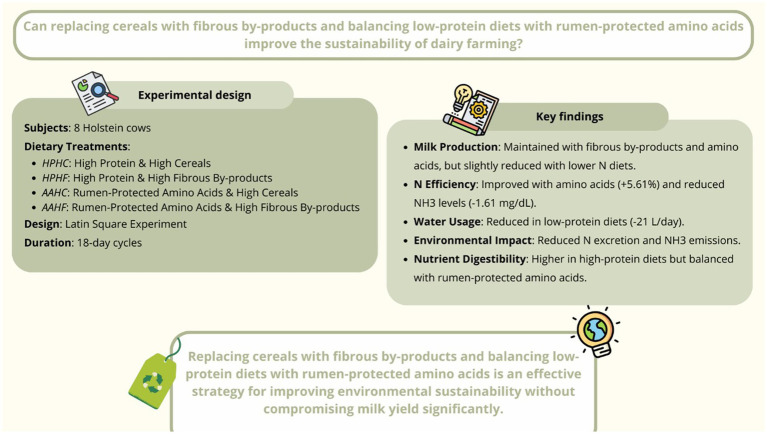

## Introduction

Finding innovative solutions to improve the sustainability of dairy farming is a crucial challenge. This requires a fresh look at established practices, particularly in feed management. Two promising strategies are the strategic use of agricultural by-products and the reduction of dietary protein—both of which can help minimize the environmental impact of dairy farms. In fact, it is well known that the balance of nitrogen (N), aminoacids and energy sources for rumen populations and dairy cows are complex processes of increasing interest in recent years (1–4).

Rumen microorganisms derive their energy supply from three main sources: degradable sugars, starch, and fiber. Sugars are generally provided by molasses, starches by cereals such as corn, wheat, sorghum, or barley, while fiber originates from forages and fibrous by-products including beet pulp, soy hulls, and cereal bran (5, 6). The reliance on cereals is effective in supporting microbial fermentation but raises concerns because these crops are also central to the human food chain, creating direct competition between food and feed uses (7–9). In contrast, fibrous by-products are mainly generated through agro-industrial processing and therefore represent resources that do not compete with human nutrition. Their inclusion in ruminant diets has been associated with the maintenance of milk yield and composition, even when cereals are partially replaced (10).

From a nutritional perspective, these by-products are particularly interesting because the potentially digestible fraction of neutral detergent fiber (pdNDF) exhibits ruminal fermentation patterns similar to those of non-structural carbohydrates, allowing them to act as functional substitutes for starch-rich feeds (11, 12). However, the use of easily fermentable fibers is not without complexity. Their degradation produces short-chain fatty acids (SCFAs), such as acetate, propionate, and butyrate, which are key energy sources for the cow but can vary in profile depending on the substrate and fermentation rate. These differences influence not only microbial growth efficiency but also systemic energy metabolism and milk composition. For this reason, the substitution of cereals with fibrous by-products must be evaluated not only in terms of feed resource sustainability but also with respect to rumen fermentation dynamics and the resulting metabolic outcomes for the animal. The N used by microorganisms to synthesize microbial proteins is mainly ammonia (NH_3_) formed in the rumen by the hydrolysis of the degradable N fraction of feeds (13). To maximize the rumen microorganism’s growth peptides, aminoacids and isoacids are also required (14).

In dairy cows, only about 25–35% of the N consumed is secreted as milk protein (15). When rumen-degraded protein (RDP) exceeds the requirements of rumen microbes, or when degradable energy for microbial growth is limiting, the efficiency of N utilization decreases sharply (16). Under such conditions, excess NH₃ produced in the rumen is absorbed into the bloodstream and converted into urea in the liver. This urea can be recycled back into the rumen via saliva and diffusion through the rumen wall, or excreted through the kidneys in urine and through the mammary gland in milk (17). The majority of urinary N is excreted in the form of urea (18), which is rapidly converted into NH₃ once in manure, thereby contributing to gaseous emissions from livestock facilities. At the whole-farm level, this process results in a significant nitrogen surplus—defined as the difference between N imported and N exported with milk, meat, or manure—which exacerbates the environmental footprint of dairy production (16).

To avoid underfeeding protein and potential losses in milk yield, producers often formulate rations with high levels of metabolizable protein. However, increasing dietary protein content also increases the supply of RDP, which not only reduces N-use efficiency but also raises feed costs due to the relatively high price of conventional protein supplements (19). A promising alternative is the adoption of low-protein diets strategically supplemented with essential rumen-protected amino acids (RP-AA). Several long-term studies have demonstrated that the inclusion of limiting amino acids such as rumen-protected methionine and lysine can maintain or even increase milk yield and milk protein synthesis (20–22). This approach improves the apparent efficiency of nitrogen utilization, expressed as the ratio of milk N to dietary N intake (18, 23), while simultaneously reducing urinary N excretion and its associated environmental burden (19).

In light of these premises, this study aims to evaluate the impact of reducing dietary crude protein levels, supplemented with rumen-protected amino acids, and replacing cereals with fibrous by-products on the sustainability of dairy farming. Specifically, it hypothesizes that these nutritional strategies will improve nitrogen utilization efficiency, reduce environmental nitrogen excretion and water consumption, while maintaining adequate milk production and rumen function.

## Materials and methods

This study did not entail any manipulation or alteration of the standard handling procedures for the animals. All cows involved in the present research were housed in a naturally ventilated individual tie-stall cubicles, enabling natural activities such as standing, lying, eating, and ruminating. All procedures were conducted in compliance with Directive 2010/63/EU, and the animals were raised in accordance with the European Union’s legal requirements (98/58/EC) concerning the protection of animals kept for farming purposes. All procedures that included animals were approved by the University of Bologna Institutional Animal Care and Use Committee (Prot. N. 4,405).

### Animals, housing, management and experimental design

The trial was conducted at the educational-experimental barn of the University of Bologna and included eight multiparous Holstein cows selected based on lactation number (2.63 ± 0.25), milk production (44.79 ± 3.30 kg), and days in milk (53.00 ± 6.49). The cows were housed in tie stalls and divided into four pairs according to the procedure reported in the study by Cavallini et al. (24). The tie stalls were equipped with individual feed troughs with continuous weighing systems and a push-button water bowl with an individual meter. Milking was performed using conventional milking system twice a day at 7:00 a.m. and 7:30 p.m.

The experiment was conducted using a Latin square experimental design. Each pair was randomly assigned to one of four isoenergetic diets in succession. The four experimental diets were identified as:

- High Protein and High Cereals—HP-HC (Control group)
- High Protein and High Fibrous By-products—HP-HF
- Rumen-Protected Amino Acids and High Cereals—AA-HC
- Rumen-Protected Amino Acids and High Fibrous By-products—AA-HF

The rations were formulated by the NDS software (RUM&N Sas, Reggio Emilia, Italy; Table 1) using hays (alfalfa and wheat) with balanced diets in protein-amino acid content and variable carbohydrate sources depending on the experimental diets (Table 1), we selected to use beet pulp, soybean hulls, and wheat bran as fibrous by-products due to their high availability and reduced cost in the context of Northern Italy. The inputs used in the software were the cows’ characteristics. Each diet was prepared individually approximately every 12 days. Daily intake was assessed based on the amount of residue present in the trough 24 h after feeding, and the amount of total mixed ration (TMR) to be provided the following day was determined to ensure 10% residue and ad libitum feeding and were fed once a day at 7:00 p.m. (Zago mixer, Padova, Italy). Additionally, each cow was offered 1 kg of long wheat hay per day according to Heinrichs et al. (25) in a separate side of the feed bunk to permit separate intake measurament.

**Table 1:** Composition and analysis of the different experimental diets administered along the trial and composition of the experimental TMR (mean data ± S. D.).

Ingredients, kg	HP-HC	HP-HF	AA-HC	AA-HF
Alfalfa hay	9.0	9.0	9.0	9.0
Wheat hay	3.0	3.0	3.0	3.0
Wheat Straw	1.0	1.0	1.0	1.0
Fibrous by-products mix^1^	1.5	5.0	3.4	7.0
Cereal mix^2^	10.0	7.0	10.0	6.5
Extruded soy extracted meal	2.5	2.0	0.6	0.5
Mineral and vitamin supplement^3^	0.5	0.5	0.5	0.5
RP Methionine	0.015	0.015	0.022	0.022
RP Lysine	-	-	0.100	0.100
Chemical composition, %				
DM intake, %	89.35 ± 1.22	88.10 ± 2.36	87.72 ± 2.81	89.90 ± 0.91
CP, % DM	14.80 ± 0.47	14.84 ± 0.91	12.47 ± 0.94	12.24 ± 1.02
Starch, % DM	26.84 ± 4.50	19.65 ± 1.57	25.86 ± 3.86	19.77 ± 2.52
aNDFom, % DM	36.40 ± 1.89	39.78 ± 2.79	38.24 ± 3.60	42.94 ± 2.30
ADF, % DM	26.25 ± 1.21	28.47 ± 2.18	26.82 ± 1.93	29.05 ± 1.13
ADL, % DM	5.37 ± 0.28	5.52 ± 0.47	5.48 ± 0.34	5.83 ± 0.52
uNDF 24 h, % DM	19.58 ± 2.42	22.69 ± 3.89	19.15 ± 2.00	23.61 ± 2.40
uNDF 240 h, % DM	12.23 ± 3.83	14.28 ± 2.12	12.12 ± 4.38	14.76 ± 3.22
peNDF	19.72 ± 1.72	21.60 ± 2.74	20.14 ± 1.90	21.41 ± 1.22
peuNDF	6.96 ± 0.68	7.30 ± 0.63	7.27 ± 0.96	7.86 ± 0.86

HP, High Protein; AA, Rumen-Protected Amino Acids; HC, High Cereals; HF, High Fibrous By-products; RP, Rumen Protected; DM, Dry Matter; CP, Crude Protein; aNDFom, Neutral Detergent Fiber; ADF, Acid Detergent Fiber; ADL, Acid Detergent Lignin; uNDF24, Undigested NDF via long-term 24 h in vitro fermentation; uNDF240, Undigested NDF via long-term 240 h in vitro fermentation; peNDF, Phisically Effective NDF; peuNDF, Phisically Effective uNDF240. Vitamins & oligo minerals* (%): 10.0. *Contributions in vitamins, provitamins, and substances with analogous effects per kg of feed: Vitamin A prov. E672 IU 40000; Vitamin D3 E671 IU 4000; Vitamin E α-tocopherol 92% prov. mg 30; Vitamin B1 mg 5; Vitamin B2 mg 3; Vitamin B6 mg 1.5; Vitamin B12 mg 0.06; Vitamin K mg 5; Vitamin H1 mg 5; Vitamin PP mg 150; Choline chloride mg 50.Contributions in trace elements per kg of feed: Iron (ferrous carbonate) E1 mg 100; Cobalt (basic cobalt carbonate) E3 mg 1; Iodine (calcium iodate anhydrous) E2 mg 5; Manganese (manganous oxide) E5 mg 120; Copper (copper sulfate pentahydrate) E4 mg 10; Zinc (zinc oxide) E6 mg 130.

1 Fibrous by-products mix (%): Beet pulp, 33.33; Soybean hulls, 33.33; Wheat bran, 33.33.

2 Cereal mix (%): Corn, 50.0; Sorghum, 50.0.

3 Mineral and vitamin supplement (%): Calcium carbonate, 23.0; Sodium chloride, 23.0; Dicalcium phosphate, 35.0; Magnesium oxide, 10.0.

### Data collection, sampling procedure and analysis

Each experimental period consisted of 14 days of adaptation (to avoid carry over effect) and 4 experimental days during which samples were collected according to the schedule shown in Table 2.

**Table 2:** Samplings during the experimental periods.

Sample	Day 15		Day 16		Day 17		Day 18	
	Evening	Morning	Evening	Morning	Evening	Morning	Evening	Morning
Feces	X	X					X	X
Milk	X	X	X	X	X	X	X	X
Rumen fluid	X	X					X	X
Blood								X

Body weight (BW) was recorded automatically after each milking by a digital scale (TDM, Brescia, Italy), while body condition score (BCS) was measured individually in the last day of each experimental period, according to Buonaiuto et al. (26).

The TMR was sampled at each new batch and raw materials were monitored throughout the experimental period to maintain consistent TMR quality. Intakes and behaviors were monitored (27).

Feces were collected from the ampulla and dried in oven at 65°C until constant weight, followed by grinding with a Cyclotec to obtain a sample particle size of 1 mm. TMR and fecal samples were analyzed for chemical composition by wet chemistry according to the following methods: crude protein (CP) according to AOAC (method 976.06 and 984.13) using a Kjeldahl nitrogen analyzer (Gerhadt Vapodest 50, Gerhardt GmbH, Königswinter, Germany), starch determined according to Ehrman and AOAC (method 920.40), ether extract according to AOAC (method 920.390020), ash-corrected *α*-amylase–treated neutral detergent fiber (NDF) with the addition of sodium sulfite (aNDFom), acid detergent fiber (ADF) and acid detergent lignin (ADL) according to Mertens et al. (28), ashed after 4 h combustion in a muffle furnace at 550°C (Vulcan 3–550, Dentsply Neytech, Burlington, NJ, United States). The ration particle size was determined using a RoTap Separator (W. S. Tyler, Mentor, OH), and the physically effective NDF (peNDF) was calculated as the product of aNDFom content and its physically effective factor (pef; (29)), whereas physically effective uNDF240h was calculated as the product of pef and uNDF240 (30). The undigested NDF at 24 h (uNDF24) and 240 h (uNDF240) and the potentially digestible NDF (pdNDF) of the ration were determined using an *in vitro* fermentation method in buffered media containing ruminal fluid and the uNDF240h was used as a marker to calculate the total tract apparent digestibility (TTD) of nutrients, as reported in Cavallini et al. (31).

The rumination time was recorded daily for each cow using the RuminAct system (SCR® – Israel) placed on a ruminometer collar (32).

The quantitative and qualitative analysis of milk was done individually for each cow and for each milking. Milk samples for analysis were taken during milking with an auto-sampler; the milk aliquot was obtained after homogenization. The milk was then poured into the container destined for analysis. The samples were obtained by mixing 50% of the milk from the evening milking with that from the following morning, stored at refrigeration temperature (+4°C), and sent to the certified laboratory within a few hours. Quality monitoring was performed on individual milk samples four times during the experimental period, and the following were analyzed: fat content, protein, lactose, somatic cell count, and urea in a certified laboratory (Milkoscan, Foss, Granlatte, Granarolo, Italy). Finally, ECM was calculated as reported in previous works (24).

Cows were monitored for reticular pH with an indwelling wireless transmitting unit (SmaXtec Animal Care Sales GmbH, Graz, Austria), a system previously validated in rumen-cannulated dairy cows (33). These devices (3.5 cm i.d., 12 cm long, and weighing 210 g) were calibrated following the manufacturer instructions and manually inserted into the rumen via the esophagus 1 week before the start of the trial. Previous research has showed that these devices tend to sit in the ventral reticulum area (33). pH and temperature were recorded every 10 min and data transmitted real-time to a base station using the ISM band (433 MHz). Data were then collected using an analog-to-digital converter and stored in an external memory chip. Reticular pH data were aggregated as daily means, and a pH threshold of 5.5 was used to calculate time and dispersion below that threshold (34).

Rumen fluid was collected via esophageal probe. The first 500 mL of rumen fluid collected was discarded before taking samples. The collected liquid was placed in 15 mL Falcon tubes and frozen pending subsequent analyses; it was then transported to the DIMEVET department laboratory. Before analysis the rumen fluid was centrifugated. Volatile fatty acid (VFA) concentrations were determined by Gas Chromatography (35), whereas NH_3_ was evaluated via commercial kit (urea/BUN—color, BioSystems S. A. Barcelona, Spain) according to the producer procedure.

Blood samples were taken from the coccygeal vein in vacuum tubes containing a clot activator (Vacutest Kima, Padova, Italy); they were then maintained at 20°C for 1 h after collection and centrifuged at 2,000 × g for 20 min to obtain the serum (Centrifugette 4,203, ALC International Srl, Cologno Monzese, Italy). Urea and uric acid were measured as reported in previous papers (36) using ADVIA 2120 (Siemens Healthcare Diagnostics).

### Statistical analysis

All data were analyzed using JMP pro v 14.3 programs. A mixed model was used for the analysis with the effect of N source (HP *vs* AA), energy source (HC *vs* HF), and their interaction as fixed effects, the individual cow (within squares and period) as a random effect (experimental unit, *N* = 8), and repeated measures for each parameter considered. Normal distribution of the data was checked for the residuals resulted from an initial mixed model, and normalized, when necessary, by BoxCox transformation. Means are reported as least square mean and pairwise multiple comparisons were performed using Student t-test as *post-hoc* test when a *p*-value ≤ 0.05 was detected. A *p*-value ≤ 0.05 was considered statistically significant; and ap-value ≤ 0.01 was considered highly significant.

## Results

The present trial showed interesting results, revealing differences related to the different protein, amino acid, and cereal and by-product content of the rations, while no differences were found for the following parameters: DMI, TMR intake, Long hay intake, DMI from concentrates, DMI from forage, OM intake, peNDF intake, Daily rumination time and Rumination/peNDF intake, Rumination/peuNDF intake, Rumination/aNDFom from forage ingested, butyrate, r-pH, r-pH < 6 min, r-pH < 6 AUC, r-pH < 6 AUC DS, r-pH < 5.8 AUC DS, r-pH < 5.5 min, r-pH < 5.5 AUC, r-pH < 5.5 AUC DS, fecal DM, fecal ADL, fecal uNDF24, Milk fat, Milk fat/protein, Milk lactose, Water intake/milk yield, Water intake/ECM, Water intake/FCM 4%, Milk/DM intake, ECM/DM intake.

Subjects fed a diet with amino acid integration showed a higher body condition score compared to those with HP diet; the recorded values were 2.37^B^, 2.41^AB^, 2.47^A^, and 2.43^AB^ for HP-HC, HP-HF, AA-HC, and AA-HF diets, respectively (*p* < 0.01). Regarding body weight, the recorded values were 639.96, 645.64, 646.87, and 641.43 kg for the HP-HC, HP-HF, AA-HC, and AA-HF diets, respectively (*p* < 0.01), although the difference between the various diets in these two parameters was negligible.

In the following paragraph are reported results regarding the Table 3. No significant differences were found in total DMI and rumination time among the animals across the four diets; the average intake was 26.40 kg. Indeed, regarding nutrient intake, a good match was observed between the expected and actual nutrient intake, confirming the accuracy of the dietary formulations used. Specifically, starch intake was 6.74^A^, 5.25^C^, 5.97^B^, and 5.14^C^ kg in the HP-HC, HP-HF, AA-HC, and AA-HF diets, respectively (*p* < 0.01), with a higher content in the HP-HC; crude protein intake was higher in HP diets (+0.79 kg, *p* < 0.01). Regarding fibrous fractions, the intake of aNDFom, ADF, and ADL was significantly higher in HF diets (+1.4 kg, +0.91, +0.12 respectively, *p* < 0.01; Table 3). The intake of uNDF24 was higher in the AA-HF diet (6.39^A^, 5.80^B^, 5.09^C^, and 5.05^C^ in the AA-HF, HP-HF, HP-HC, and AA-HC diets respectively; *p* < 0.01). The intake of uNDF240 and peuNDF was higher in HF diets compared to HC diets (+0.57 kg and +0.13 kg respectively, *p* < 0.01). Additionally, higher daily water intake (HP = 167.88 L vs. AA = 146.98 L) was observed in high-protein diets.

**Table 3:** Nutrient intake, and water intake based on different protein, synthetic amino acid, cereal, and fibrous by-product levels.

Item	HP		AA		SEM	*p-*value		
	HC	HF	HC	HF		HP vs. AA	HC vs. HF	Interaction
DMI, kg	26.55	26.99	25.49	26.55	0.76	0.06	0.06	0.44
TMR, kg	25.81	26.05	24.58	25.66	0.75	0.05	0.10	0.30
Long hay, kg	0.74	0.94	0.92	0.88	0.17	0.43	0.29	0.12
DMI from concentrates, kg	13.56	13.68	12.96	13.54	0.40	0.08	0.10	0.27
DMI from forage, kg	13.00	13.31	12.53	13.01	0.40	0.05	0.04	0.70
OM intake, kg DM	24.79	25.24	23.78	24.97	0.65	0.11	0.04	0.35
Starch intake, kg DM	6.74^A^	5.25^C^	5.97^B^	5.14^C^	0.18	<0.01	<0.01	<0.01
CP intake, kg DM	4.10	4.22	3.31	3.42	0.10	<0.01	0.08	0.98
aNDFom intake, kg DM	9.59	10.76	9.92	11.55	0.27	<0.01	<0.01	0.20
ADF intake, kg DM	7.07	7.86	7.09	8.12	0.19	0.29	<0.01	0.36
ADL intake, kg DM	1.46	1.55	1.46	1.60	0.04	0.32	<0.01	0.24
uNDF24 intake, kg DM	5.09^C^	5.80^B^	5.05^C^	6.39^A^	0.19	<0.01	<0.01	<0.01
uNDF240 intake, kg DM	3.52^C^	3.90^B^	3.66^BC^	4.41^A^	0.11	<0.01	<0.01	0.02
peNDF intake, kg DM	5.79	6.06	6.19	6.27	0.21	0.03	0.20	0.52
peuNDF intake, kg DM	2.00	2.06	2.08	2.27	0.06	<0.01	<0.01	0.15
Water intake, L	170.41	165.34	141.43	152.53	11.92	<0.01	0.62	0.19

^A–C^*p* < 0.05; HP, High Protein; AA, Rumen-Protected Amino Acids; HC, High Cereals; HF, High Fibrous By-products; DMI, Dry Matter Intake; TMR, Total Mixed Ration; OM, Organic Matter; CP, Crude Protein; aNDFom, Neutral Detergent Fiber; ADF, Acid Detergent Fiber; ADL, Acid Detergent Lignin; uNDF24, Undigested NDF via long-term 24 h in vitro fermentation; uNDF240, Undigested NDF via long-term 240 h in vitro fermentation; peNDF, Phisically Effective NDF; peuNDF, Phisically Effective uNDF240.

In Table 4 are reported the data regarding the rumen environment and behavior. The average rumination time was 506 min/day during the trial and HF cows ruminated 22 min/day on average less (*p* = 0.03). Cows receiving HC diets showed a significant increase in the time spent to ruminate per unit of aNDFom (HC = 51.39 min/kg vs. HF = 46.76 min/kg, *p* < 0.01). As well as for the results of rumen fluid, a significant difference in the amount of NH_3_ measured was found between P diets and those with amino acid integration; the latter are associated with lower content compared to HP diets (AA = 3.33 mg/dL vs. HP = 4.94 mg/dL, *p* < 0.01). Furthermore, this parameter is higher in HF diets compared to HC diets (HF = 4.45 mg/dL vs. HC = 3.82 mg/dL, *p* < 0.01).

**Table 4:** Rumination times, rumen fluid analysis, and reticulorumen pH values based on different protein, synthetic amino acid, cereal, and fibrous by-product levels.

Item	HP		AA		SEM	*p-*value		
	HC	HF	HC	HF		HP vs. AA	HC vs. HF	Interaction
Rumination, min	503.31	515.06	487.84	518.63	18.00	0.53	0.03	0.31
Rumination/DM intake, min/kg	19.05	19.26	19.35	19.65	0.80	0.42	0.55	0.93
Rumination/aNDFom intake, min/kg	52.72	48.33	50.06	45.18	1.78	0.02	<0.01	0.84
Rumination/peNDF intake, min/kg	87.84	86.75	82.13	83.25	4.04	0.05	0.99	0.64
Rumination/peuNDF intake, min/kg	253.82	252.70	240.81	231.35	8.92	0.01	0.44	0.54
Rumination/aNDFom from forage ingested, min/kg	85.16	85.25	86.15	87.64	3.22	0.40	0.69	0.72
NH_3_, mg/dl	4.81^A^	5.06^A^	2.82^C^	3.84^B^	0.57	<0.01	<0.01	0.04
VFA, mmol/L	90.85	84.01	89.89	86.96	5.03	0.55	<0.01	0.24
Acetate, %/mmol	57.75	59.29	58.18	59.73	1.27	0.03	<0.01	0.98
Propionate, %/mmol	26.55	25.44	26.68	25.49	1.49	0.74	<0.01	0.88
Iso-butyrate, %/mmol	0.88	0.83	0.80	0.80	0.03	<0.01	0.27	0.12
Nor-butyrate, %/mmol	12.03	11.90	11.91	11.56	0.69	0.02	0.02	0.26
Total-butyrate, %/mmol	12.90	12.73	12.70	12.36	0.70	<0.01	0.02	0.41
Iso-valeriate, %/mmol	1.25^A^	1.09^B^	1.02^BC^	0.99^C^	0.09	<0.01	<0.01	0.02
Nor-valeriate, %/mmol	1.55^A^	1.45^B^	1.42^B^	1.43^B^	0.07	<0.01	0.03	<0.01
Total-valeriate, %/mmol	2.80^A^	2.54^B^	2.43^B^	2.42^B^	0.11	<0.01	<0.01	<0.01
Acetate: Propionate	2.26	2.39	2.25	2.40	0.16	0.94	<0.01	0.71
Nor-Butyrate: Propionate	0.47	0.48	0.47	0.46	0.04	0.15	0.78	0.54
Acetate+Nor-Butyrate: Propionate	2.73	2.87	2.72	2.86	0.19	0.86	<0.01	0.97
r-pH	6.06	6.04	6.08	6.03	0.06	0.71	<0.01	0.13
r-pH, DS	0.17	0.16	0.15	0.14	0.02	0.02	0.34	0.34
r-pH > 6.3, min	212.50	107.50	207.98	133.13	81.41	0.65	<0.01	0.51
r-pH > 6.3, AUC	34.29	16.65	33.99	14.58	17.43	0.83	<0.01	0.88
r-pH > 6.3, AUC DS	0.40	0.26	0.33	0.20	0.16	0.11	<0.01	0.79
r-pH > 6.1, min	645.94	567.5	673.49	543.75	137.20	0.96	<0.01	0.50
r-pH > 6.1, AUC	117.4	75.77	116.59	78.94	36.74	0.90	<0.01	0.84
r-pH > 6.1, AUC DS	0.87	0.72	0.76	0.64	0.21	0.04	<0.01	0.80
r-pH < 6, min	533.75	549.38	491.53	590.63	156.28	0.99	0.12	0.26
r-pH < 6, AUC	111.28^A^	89.48^AB^	79.45^B^	109.87^A^	41.84	0.57	0.67	0.01
r-pH < 6, AUC DS	0.73	0.74	0.63	0.74	0.19	0.36	0.23	0.35
r-pH < 5.8, min	246.56^A^	178.44^AB^	138.44^B^	213.13^AB^	99.83	0.15	0.89	<0.01
r-pH < 5.8, AUC	33.41^A^	19.51^AB^	17.41^B^	33.23^A^	15.26	0.85	0.88	<0.01
r-pH < 5.8, AUC DS	0.36^A^	0.28^AB^	0.23^B^	0.35^A^	0.14	0.54	0.67	0.03
r-pH < 5.5, min	17.19	8.13	8.30	20.94	10.91	0.77	0.79	0.08
r-pH < 5.5, AUC	1.06^AB^	0.77^B^	0.79^AB^	3.41^A^	1.61	0.36	0.37	0.03
r-pH < 5.5, AUC DS	0.036	0.024	0.020	0.068	0.03	0.57	0.47	0.08

^A–C^*p* < 0.05; HP, High Protein; AA, Rumen-Protected Amino Acids; HC, High Cereals; HF, High Fibrous By-products; DM, Dry Matter; aNDFom, Neutral Detergent Fiber; peNDF, Phisically Effective NDF; peuNDF, Phisically Effective uNDF240; VFA, Volatile Fatty Acids; SD, Standard Deviations; AUC, Area Under Curve.

Overall, total VFA were found in lower percentages in rumen fluid with HF diets (HF = 85.49 mmol/L vs. HC = 90.37 mmol/L, *p* < 0.01) with a higher acetate in HF diets (HF = 59.51%/mmol vs. HC = 57.97%/mmol, *p* < 0.01) and propionate in HC diets (HC = 26.62%/mmol vs. HF = 25.47%/mmol, *p* < 0.01); the acetate to propionate ratio was statistically higher in HF diets (HF = 2.40 vs. HC = 2.26, *p* < 0.01) as well as the acetate plus nor-butyrate to propionate ratio (HF = 2.87 vs. HC = 2.73, *p* < 0.01). Iso-butyrate was measured in higher percentage in HP diets (HP = 0.86%/mmol vs. AA = 0.80%/mmol, *p* < 0.01) as well as the total butyrate content (HP = 12.82%/mmol vs. AA = 12.53%/mmol, *p* < 0.01). Iso-valerate was measured with higher values in HP-HC, HP-HF, AA-HC, and AA-HF diets respectively: 1.25^A^, 1.09^B^, 1.02^BC^, and 0.99^C^ (*p* < 0.01). Nor-valerate was measured in higher %/mmol in HP-HC, HP-HF, AA-HC, and AA-HF diets respectively: 1.55^A^, 1.45^B^, 1.42^B^, and 1.43^B^ (*p* < 0.01), as well as iso-valerate + nor-valerate (HP-HC = 2.80^A^, HP-HF = 2.54^B^, AA-HC = 2.43^B^, and AA-HF = 2.42^B^, *p* < 0.01; Table 4).

Observing the reticulorumen pH values reported in Table 4, it is noted that HC diets have statistically higher daily values compared to HF diets (HC = 6.07 vs. HF = 6.04, *p* < 0.01) with higher levels of r-pH above the threshold of 6.3 (HC = 210.24 min vs. HF = 120.32 min, HC = 34.14 AUC vs. HF = 15.62 AUC, HC = 0.37 AUC DS vs. HF = 0.23 AUC DS, *p* < 0.01) and above the threshold of 6.1 (HC = 659.72 min vs. HF = 555.63 min, HC = 117.0 AUC vs. HF = 77.36 AUC, HC = 0.82 AUC DS vs. HF = 0.68 AUC DS, *p* < 0.01). The time in which r-pH was recorded below the value of 5.8 was 246.56A, 178.44AB, 138.44B, 213.13AB min for the HP-HC, HP-HF, AA-HC, and AA-HF diets, respectively (*p* < 0.01) with an AUC of 33.41A, 19.51AB, 17.41B, 33.23A, respectively (*p* < 0.01).

Serum urea concentrations increased with increasing N intake, thus higher in HP diets with values of 25.76, 27.14, 17.73, and 19.11 in the HP-HC, HP-HF, AA-HC, and AA-HF diets, respectively (*p* < 0.01) as well as in the case of uric acid (HP-HC = 0.97, HP-HF = 0.94, AA-HC = 0.76, and AA-HF = 0.84, *p* < 0.01).

Concerning fecal analysis and nutrient digestibility reported in Table 5, pH was statistically higher in HP diets compared to AA diets (HP = 6.35 vs. AA = 6.30, *p* < 0.01). Regarding composition, CP content was higher in HP diets (HP = 14.62% vs. HC = 13.91%, *p* < 0.01); while in AA diet, starch (AA = 3.29% vs. HP = 2.92%, *p* < 0.01), aNDFom (AA = 61.99% vs. HP = 60.45%, *p* < 0.01), and ADF (AA = 46.21% vs. HP = 45.36%, *p* < 0.01) resulted augmented. ADF was lower in HC diets compared to HF diets (HC = 45.19% vs. HF = 46.38%, *p* < 0.01) as well as ash (HC = 9.99% vs. HF = 10.26%, *p* < 0.01). uNDF_240_ was present in feces with a content of 48.79^A^, 46.76^AB^, 45.25^BC^, and 43.74^C^, in the HP-HC, AA-HF, HP-HF, and AA-HC diets, respectively (*p* < 0.01).

**Table 5:** Fecal analysis and nutrient digestibility based on different protein, synthetic amino acid, cereal, and fibrous by-product levels.

Item	HP		AA		SEM	*p-*value		
	HC	HF	HC	HF		HP vs. AA	HC vs. HF	Interaction
pH	6.33	6.36	6.31	6.28	0.03	<0.01	0.87	0.08
DM, %	13.10	13.07	12.98	12.70	0.19	0.03	0.18	0.27
Ash, %DM	9.87^B^	10.36^A^	10.09^AB^	10.15^AB^	0.18	0.93	<0.01	0.02
Starch, %DM	2.81^C^	3.03^BC^	3.34^A^	3.24^AB^	0.15	<0.01	0.32	0.02
CP, %DM	14.65^A^	14.58^A^	14.22^A^	13.60^B^	0.26	<0.01	0.03	0.08
aNDFom, %DM	60.36	60.54	61.59	62.38	0.62	<0.01	0.12	0.36
ADF, %DM	44.92	45.79	45.45	46.97	0.50	<0.01	<0.01	0.09
ADL, %DM	18.56^A^	18.47^AB^	17.80^B^	18.74^A^	0.59	0.32	0.09	0.05
uNDF_24_, %DM	53.49	53.15	53.37	53.73	0.51	0.36	0.97	0.18
uNDF_240_, %DM	48.79^A^	45.25^BC^	43.74^C^	46.76^AB^	1.45	<0.01	0.65	<0.01
TTOMD, %OM	73.19	70.11	68.18	67.17	1.16	<0.01	<0.01	0.17
TTstarchD, %Starch	96.91	95.15	95.36	94.12	0.37	<0.01	<0.01	0.11
TTCPD, %CP	73.37	70.72	63.96	64.08	1.58	<0.01	0.20	0.18
TTaNDFomD, %aNDFom	54.35	52.59	48.50	49.05	1.46	<0.01	0.57	0.30
TTADFD, %ADF	54.01	51.29	46.73	46.14	1.59	<0.01	0.15	0.37
TTpdNDF24D, %pdNDF24	88.72	87.22	85.75	83.37	0.64	<0.01	<0.01	0.29
TTpdNDF240D, %pdNDF240	83.20^A^	78.24^B^	71.44^C^	76.81^B^	2.18	<0.01	0.87	<0.01

^A–C^*p* < 0.05; HP, High Protein; AA, Rumen-Protected Amino Acids; HC, High Cereals; HF, High Fibrous By products; TTD, Total-Tract Digestibility; DM, Dry Matter; OM, Organic Matter; CP, Crude Protein; aNDFom, Neutral Detergent Fiber; ADF, Acid Detergent Fiber; ADL, Acid Detergent Lignin; uNDF24, Undigested NDF after 24 h in vitro fermentation; uNDF240, Undigested NDF after 240 h in vitro fermentation;pdNDF24, Potentially Degradable NDF after 24 h in vitro fermentation; pdNDF240, Potentially Degradable NDF after 240 h in vitro fermentation.

The TTOMD (% OM) was significantly higher in HP diets (HP = 71.65% vs. AA = 67.68%, *p* < 0.01) and in HC diets (HC = 70.69% vs. HF = 68.64%, *p* < 0.01) as well as TTstarchOD (HP = 96.03% vs. AA = 94.74%, HC = 96.14% vs. HF = 94.64%, *p* < 0.01) and TTpdNDF24D (HP = 87.97% vs. AA = 84.56%, HC = 87.24% vs. HF = 85.30%, *p* < 0.01). TTCPD was also higher in HP diets (HP = 72.05% vs. AA = 64.02%, *p* < 0.01), TTaNDFomD (HP = 53.47% vs. AA = 48.78%, *p* < 0.01), and TTADFD (HP = 52.65% vs. AA = 46.44%, *p* < 0.01). TTpdNDF_240_D resulted significantly influenced by the dietary treatments 83.20^A^, 78.24^B^, 71.44^C^, and 76.81^B^ in the HP-HC, HP-HF, AA-HC, and AA-HF diets, respectively (*p* < 0.01).

As shown in Table 6, total milk production was statistically higher in diets with higher protein content. However, the difference from other diets was at most 1.32 kg, with an average of 37.71 kg for HP diets and 36.39 kg for AA diets (*p* < 0.01). The same applies to ECM, where values of 39.18 kg and 37.44 kg were observed for HP diets and AA diets, respectively (*p* < 0.01). The milk total protein content was influenced by the diet’s energy source, with a higher percentage in HC diets; however, the difference from HF diets was minimal (HC = 3.40% vs. HF = 3.33%, *p* < 0.01). MUN levels were influenced by both N and fiber content of the administered diets, with higher amounts recorded in HP diets (HP = 12.74 mg/dL vs. AA = 8.36, *p* < 0.01) and in HF diets (HF = 10.99 mg/dL vs. HC = 10.11 mg/dL, *p* < 0.01).

**Table 6:** Quantitative and qualitative analysis of milk based on different protein, synthetic amino acid, cereal, and fibrous by-product levels.

Item	HP		AA		SEM	*p-*value		
	HC	HF	HC	HF		HP vs. AA	HC vs. FB	Interaction
Milk, kg	37.90	37.52	36.75	36.03	2.47	<0.01	0.10	0.64
ECM, kg	39.36	39.00	37.75	37.12	1.87	<0.01	0.10	0.45
Fat, %	3.60	3.63	3.60	3.57	0.15	0.48	0.94	0.57
Protein, %	3.41	3.31	3.39	3.35	0.13	0.64	<0.01	0.15
Fat/Protein	1.06	1.10	1.06	1.07	0.03	0.42	0.21	0.38
Lactose, %	4.79	4.78	4.80	4.78	0.04	0.76	0.37	0.87
MUN, mg/dl	12.23	13.25	7.99	8.72	1.07	<0.01	<0.01	0.62

HP, High Protein; AA, Rumen-Protected Amino Acids; HC, High Cereals; HF, High Fibrous By-products; ECM, Energy Corrected Milk [Milk, kg* (0.383 * % Fat + 0.242 * % Protein + 0.7832) / 3.1138]; FCM, Fat Corrected Milk (4%) = 0.4 * Milk, kg + 18.57 * % Fat; MUN, Milk Urea Nitrogen; DM, Dry Matter.

Higher water intake per unit of dry matter intake (HP = 6.36 L/kg vs. AA = 5.72 L/kg, *p* < 0.01) was observed in high-protein diets (Table 7).

**Table 7:** Efficiency indices of water, feed, and N based on different protein, synthetic amino acid, cereal, and fibrous by-product levels.

Item	HP		AA		SEM	*p-*value		
	HC	HF	HC	HF		HP vs. AA	HC vs. HF	Interaction
Water intake/DM intake, L/kg	6.48	6.24	5.68	5.75	0.53	<0.01	0.72	0.51
Water intake/milk yield, L/kg	4.59	4.50	4.13	4.29	0.44	0.05	0.83	0.43
Water intake/ECM, L/kg	4.79	4.76	4.30	4.52	0.46	0.05	0.61	0.51
N retention, %	30.60	28.91	36.47	34.27	1.21	<0.01	<0.01	0.69
MUN/ ingested nitrogen, %	0.71	0.75	0.56	0.57	0.07	<0.01	0.20	0.46
Milk/DM intake kg	1.43	1.40	1.43	1.37	0.08	0.57	0.04	0.46
ECM/DM intake, kg	1.36	1.33	1.36	1.30	0.06	0.44	0.04	0.47
MUN/DM intake, g/kg	0.08	0.09	0.05	0.05	0.01	<0.01	0.08	0.43

HP, High Protein; AA, Rumen-Protected Amino Acids; HC, High Cereals; HF, High Fibrous By-products; ECM, Energy Corrected Milk; FCM, Fat Corrected Milk (4%); MUN, Milk Urea Nitrogen; DM, Dry Matter; N retention, Protein nitrogen in milk/ingested nitrogen.

As for the percentage of dietary N converted into total milk protein, as shown in Table 7, it was higher in diets integrated with AA (AA = 35.37% vs. HP = 29.76%, *p* < 0.01) and in HC diets (HC = 33.54% vs. HF = 31.59%, *p* < 0.01). Linearly, the percentage ratio between N excreted with milk as urea and N intake is higher in HP diets compared to AA diets (HP = 0.73% vs. AA = 0.57%, *p* < 0.01) as well as the ratio between N excreted with milk as urea and DMI (HP = 0.09 g/kg vs. AA = 0.05 g/kg, *p* < 0.01).

## Discussion

The primary goal of this research was to enhance the sustainability of dairy farming by implementing a two-part strategy. The first part involved reducing the protein content of the animals’ rations, which were then balanced with synthetic amino acids. Concurrently, the second part of the strategy replaced traditional starchy energy sources from cereals with fibrous by-products. This comprehensive approach sought to identify a new feeding method that could maintain adequate milk production while simultaneously minimizing environmental impact. The ultimate objective was to demonstrate a practical way for the dairy industry to become more sustainable without compromising productivity.

Based on the obtained data, no statistically significant differences were found in DMI, TMR intake, long hay intake, DMI from concentrates, DMI from forage, OM intake, and peNDF intake. DMI was maintained at the same level across all diets, averaging around 26 kg, in line with the requirements of pluriparous dairy cows, producing over 36 kg/d of milk. Concerning milk production, the diet did not influence milk fat, fat/protein ratio, lactose, milk yield/DMI, and ECM/DMI. Similar results were observed in the research conducted by Chowdhury et al. (2), where it was noted that the dietary treatment did not affect DMI, although the average was 21.5 kg/d, lower than that found in the present study. This data confirmed that intake is more determined by forage quality, particle size, and digestibility rather than the dietary protein level (37); it is indeed reported in the literature that rations based on hays increase intake compared to iso-protein silage-based diets (38). The nutrient intake data were consistent with the expected results, confirming that the objective of having cows ingest different proportions of proteins, starches, and highly digestible fibers was achieved. Variations in the composition of the experimental diets resulted in significant differences in starch, CP, aNDFom, ADF, ADL, uNDF_24_, uNDF_240_, and peuNDF intake, confirming what was reported by Barrientos-Blanco et al. (39). The measured rumination time is considered adequate for cows in good health. This falls within the typical circadian rhythm of cattle, which normally spend 8–9 h per day ruminating (40, 41). Specifically, the average rumination time for healthy dairy cows is estimated at 463 min per day for primiparous cows and 522 min per day for pluriparous cows (41). Diet significantly influenced rumination time (HC vs. HF, *p* = 0.03), along with the rumination/peNDF (HP vs. AA, *p* = 0.05) and rumination/peuNDF (HP vs. AA, *p* = 0.01). In terms of water intake, high-protein diets differed significantly from those with amino acid supplementation, an increase in total water consumption (L) and per kg of dry matter (L/kg) was observed in diets with higher extruded soybean meal content, while water intake/milk yield, and water intake/ECM were not affected. This phenomenon can be explained by the fact that higher dietary protein intake leads to an increased production of N metabolites, which must be excreted through urine, thereby increasing its volume (42–44). This interpretation is supported by the observed increases in MUN levels, ruminal NH₃, and serum urea, which will be discussed further. This finding is consistent with the results of Leonardi *et al.* (15), who reported that high-protein diets (18.8% CP) increased estimated urine volume by about 2.9 L/day compared with low-protein diets (16.1% CP). Their study was conducted in multiparous dairy cows producing on average 47.6 ± 7.1 kg/day and in primiparous cows producing 35.2 ± 2.1 kg/day. In line with this evidence, the present study also shows that high-protein diets, although the most effective in increasing milk yield, are associated with higher MUN levels (23). This situation has negative implications not only environmentally, increasing the risk of water eutrophication (16), but also in terms of management, leading to issues related to slurry production on farms (Directive 91/676/EEC). The rumen fluid analysis data did not show differences in the percentage composition of Nor-butyrate and the Nor-Butyrate: Propionate ratio. It can be observed that cows fed high-protein diets recorded higher measured NH_3_ levels (44). This evidence is attributable to the higher amount of proteins in the rumen provided by these diets, represented by extruded soybean meal, leading to high hydrolysis of the N fraction of the feeds and, consequently, NH_3_ production. In fact, N utilization efficiency, measured by the percentage of dietary N converted into milk proteins, is higher in rumen-protected amino acid integrated diets compared to high-protein diets; this aspect indicates that these diets are more efficient in promoting N use for milk protein synthesis (2, 23). The average ruminal NH₃ concentrations observed in this study (~5 mg/dL in high-protein diets and ~3 mg/dL in diets with amino acid integration) are noteworthy in light of the long-standing benchmark of ~5 mg/dL proposed by Satter and Slyter (45) as the minimum required for maintaining optimal bacterial growth and fiber digestion. Several authors, however, have reported higher thresholds. Mehrez and Ørskov (46) observed maximal fermentation in sheep at ~23.5 mg/dL, while Hoover (47) suggested ~6.2 mg/dL and more recent reviews reported values ranging from 15 to 25 mg/dL depending on the response variable considered (microbial growth or fiber digestibility) (47, 48). In contrast, pure-culture studies indicate that predominant rumen bacteria can achieve near-maximal growth at much lower ammonia concentrations, around 1–2 mg/dL, suggesting that the requirement is not universal but strongly diet-dependent (49, 50). In this sense, the present findings challenge the assumption of a fixed minimum NH₃ concentration. Despite ruminal NH₃ levels falling below 5 mg/dL in the amino acid-supplemented group, animal performance was maintained, supporting the hypothesis that efficient synchronization between fermentable energy and nitrogen supply can reduce the need for high bulk NH₃ concentrations. The results obtained in this study show that with lower proteins levels indicate that total tract potentially digestible NDFom (TTpdNDFom) digestibility as well as milk production were reduced indicating that a lack of N in the rumen can be speculated. Especially when RDP of the diet is limited also isoacids provision can be limited and isoacids are essential to stimulate the fibrolitic bacteria activity in the rumen (51). When protein level in the diets are low the urea recirculation increased (NASEM, 2021). Diet, including the amount of RDP and feeding frequency, greatly influences the proportion of recycled urea compared to that excreted (52, 53). Urea recycling in ruminants is a fundamental process for optimizing N utilization; it allows ruminants to use urea, produced during protein catabolism, as a N source for microbial protein synthesis in the rumen, significantly contributing to reducing N loss to the environment through lower urinary urea excretion. The analysis conducted on rumen fluid also showed that cows fed high-protein diets had higher butyrate levels compared to those fed rumen-protected amino acid integrated diets. As far as we know, there are no studies in the literature that have found similar data. Based on the results from fecal analysis, the diet did not impact fecal DM, fecal ADL, and fecal uNDF24. High-protein diets had a higher residual CP percentage; this data could be attributed to the fact that a higher amount of protein can bypass the rumen without being completely digested, leading to an increase in such substance at the fecal level. The higher protein content in diets as expected led to higher nutrient digestibility; this could be attributed to the fact that the higher protein content, associated with the higher NH_3_ levels measured in the rumen fluid, provided a greater N substrate for bacterial growth (2, 44). A higher CP content in the diet (up to 22% of diet DM) has been associated with small improvements in aNDFom and pdNDF digestibility in meta-analyses (54) and in individual studies (55). Therefore, microorganisms grew more, allowing them to better digest different nutrients. This data is also confirmed by higher uric acid levels found in serum for high-protein diets; this parameter is a by-product of ruminal microbial growth and, therefore, is associated with better bacterial growth (56). Moreover, fecal pH was also observed to be higher in HP diets, hypothesized to be due to a lower amount of residual fermentable starch in the large intestine (rumen by pass starch), undergoing less fermentation and resulting in higher pH.

Concerning significant differences between high-cereal diets and those with high fibrous by-products, an interesting aspect is the rumination time per unit of aNDFom ingested. The values observed were higher in subjects receiving a diet with a higher starch content. The explanation for this result can be attributed to the difference in aNDFom between cereal-based and fibrous by-product-based diets, due to the higher amount of fibrous feeds in by-products. Forages, on the other hand, were represented equally in all four diets. This hypothesis is further supported by the fact that rumination time per unit of aNDFom derived from forages does not vary between cereal-based and fibrous by-product-based diets, confirming that it is the fibrous feed component that determines the higher amount of aNDFom. Regarding the analysis conducted on rumen fluid, acetate, and the acetate:propionate ratio was higher in high fibrous by-product diets compared to high-cereal diets, as these contain a higher proportion of aNDFom. The molar percentage of acetate and the acetate-to-propionate ratio are typically higher in cattle adapted to high-fiber diets. This outcome reflects the fact that acetate is the principal end-product of microbial fermentation of structural carbohydrates, particularly cellulose and hemicellulose, by fibrolytic bacteria in the rumen (57, 58). In contrast, diets rich in rapidly fermentable starch shift the ruminal microbial ecosystem toward amylolytic species and lactate-utilizing bacteria, favoring propionate production through the succinate and acrylate pathways (59, 60). Consequently, high-cereal diets generally increase ruminal propionate concentrations while reducing acetate proportion, which not only modifies the acetate-to-propionate ratio but also alters the supply of glucogenic versus lipogenic precursors available to the host. Concerning r-pH, no significant differences were found between the four diets, with an average value of 6.05. No differences were also observed in the following parameters: r-pH < 6 min, r-pH < 6 AUC, r-pH < 6 AUC DS, r-pH < 5.8 AUC DS, r-pH < 5.5 min, r-pH < 5.5 AUC, and r-pH < 5.5 AUC DS. The high-protein and high-cereal diet (HP-HC) was identified as the one at the highest risk of ruminal acidosis; however, during the administration of all diets, no enough time of pH below the threshold was recorded, corresponding to a value of 5.5 for 330 min/day (61). When evaluating ruminal pH dynamics, HC diets exhibited greater variability in the time spent above 6.3 and below 5.8. However, this fluctuation was not accompanied by clinical signs, was not associated with differences in long hay intake, and did not result in milk fat depression, as indicated by the stable fat-to-protein ratio. Indeed, regarding long hay intake, it was slightly below 1 kg/head per day, as what has been reported in previous studies (24, 25), confirming that animals prefer longer particle forages, which have a high ruminal buffering effect and promote rumination activity (62). Specifically, Keunen et al. (63) and Maulfair et al. (64) demonstrated that lactating cows induced with sub-acute ruminal acidosis (SARA) showed a dietary preference for higher peNDF and slower starch fermentability. Our results are consistent with those of Rivera-Chacon et al. (65), who reported that daily ruminal pH oscillations are strongly influenced by diet composition. In their study, high-cereal diets (mean starch content 28.45% ± 1.72) produced greater diurnal variation in ruminal pH compared with forage-based diets. Similarly, in the present trial, high-cereal feeding led to a wider range of pH values over time. However, in both studies these fluctuations did not reach statistical significance, suggesting that although ruminal pH variation is evident, its biological impact under the tested conditions remains limited. High-cereal diets were associated with higher pdNDF24 digestibility. This effect may be explained by the greater availability of starch, which provides additional energy for ruminal microbial growth and thus enhances overall nutrient digestion. However, when expressed as a percentage, TTNDF240D did not differ between the AA-HF and HP-HF groups. Beyond fiber utilization, high-cereal diets also improved milk composition, increasing milk protein percentage compared with the other dietary treatments. This outcome is consistent with enhanced microbial protein synthesis, as previously discussed. In agreement with this interpretation, Cavallini et al. (37) demonstrated through neural network modeling that the optimal starch inclusion for Parmigiano Reggiano production lies between 23 and 26%, a range that corresponds closely to the starch content of the HP-HC diet tested in the present study.

To conclude, reducing dietary CP, compensated using adequate supplies of critical rumen-protected amino acids, can improve N use efficiency in dairy cows and reduce N losses from excretions, decreasing environmental impact (66, 67), but at the same time, the milk production and digestibility were negatively affected. Moreover, low-protein diets are associated with lower water consumption, adding another dimension to their environmental benefits. The integration of fibrous by-products represents an effective strategy to improve the sustainability of the dairy supply chain. Several studies have demonstrated that using such waste products instead of traditional feeds can reduce agricultural land use by 35%, carbon emissions by 20%, and eutrophication potential (Lindberg et al., 2021) (8, 68, 69). Furthermore, the economic feasibility of by-products ensures that farmers can adopt such practices without compromising productivity or profitability. Another significant advantage concerns the reduction of competition for food resources between livestock production and human consumption; by using fibrous by-products, which are not intended for human consumption, more cereals and other resources are available for feeding people, contributing to global food security. Future studies should continue to evaluate the long-term impacts of using by-products in dairy diets while optimizing supply chains and existing processing techniques to further enhance their economic and environmental benefits.

This study, while making a significant contribution to research on nutrition and sustainability in dairy farming, has some limitations. Firstly, the latin square experimental design involved four experimental periods of only 18 days, a too-short time frame to deepen the effect of diets on the body condition and muscle mass of the animals. These parameters were not influenced by the administered diet, preventing the determination of whether, over a longer period, significant impacts could emerge. Furthermore, the population size analyzed was limited. In light of these findings, future perspectives including the analysis of a larger sample over a more extended experimental period will allow more knowledges to better refine the rations guidelines for dairy cows.

## Conclusion

Lowering the protein content in cattle diets to around 12% and supplementing it with rumen-protected amino acids is an effective way to improve N use efficiency. This approach leads to a reduction in N losses through milk and excretions, which is beneficial for the environment. Additionally, these low-protein diets have the added benefit of reducing the animal’s water consumption.

However, this strategy is not without its limitations. If the ruminal N supply becomes too low, it can negatively impact milk production and diet digestibility, potentially because of a lack of essential isoacids.

To address this, another viable strategy is to replace some of the cereals in the diet with high-fiber by-products. This substitution does not negatively affect the animals’ DMI, rumination time, or milk production and quality. This not only offers a valid alternative to high-cereal diets but also reduces the competition for food resources between livestock and humans.

Future research should focus on the long-term effects of these dietary changes to fully optimize their environmental and economic benefits.

## Data Availability

The raw data supporting the conclusions of this article will be made available by the authors, without undue reservation.
